# Sitagliptin affects gastric cancer cells proliferation by suppressing Melanoma‐associated antigen‐A3 expression through Yes‐associated protein inactivation

**DOI:** 10.1002/cam4.3024

**Published:** 2020-03-30

**Authors:** Qi Wang, Pan Lu, Tao Wang, Qianqian Zheng, Yan Li, Sean X. Leng, Xin Meng, Biao Wang, Jisheng Xie, Haiyan Zhang

**Affiliations:** ^1^ Department of Geriatrics The First Affiliated Hospital of China Medical University Shenyang Liaoning China; ^2^ Department of Urinary Surgery The Central Hospital of Xiaogan Xiaogan Hubei China; ^3^ Department of Pathology Shenyang KingMed Center for Clinical Laboratory Co., Ltd. Shenyang Liaoning China; ^4^ Department of Pathophysiology College of Basic Medical Science China Medical University Shenyang Liaoning China; ^5^ Department of General Surgery The Fourth Affiliated Hospital of China Medical University Shenyang Liaoning China; ^6^ Division of Geriatric Medicine and Gerontology Department of Medicine Johns Hopkins University School of Medicine Baltimore MD USA; ^7^ Department of Biochemistry and Molecular Biology School of Life Sciences China Medical University Shenyang Liaoning China; ^8^ Department of Histology and Embryology Youjiang Medical College for Nationalities Baise Guangxi China

**Keywords:** gastric cancer, MAGE‐A3, sitagliptin, YAP

## Abstract

Sitagliptin is an emerging oral hypoglycemic agent that inhibits the development of a wide variety of tumors. Current researches indicate that the abnormal activation of Yes‐associated protein (YAP) promotes the proliferation and poor prognosis of multiple tumors. However, the ability of sitagliptin to regulate YAP and its effects on gastric cancer (GC) cells remain unclear. Here, we first showed that sitagliptin inhibited the proliferation of GC cells, and this inhibition was regulated by Hippo pathway. Sitagliptin phosphorylated YAP in a large tumor suppressor homolog‐dependent manner, thereby inhibiting YAP nuclear translocation, and promoted YAP cytoplasm retention. This inhibition can be blocked by adenosine 5′‐monophosphate‐activated protein kinase (AMPK). Moreover, sitagliptin could reduce the expression of tumor‐testis antigen Melanoma‐associated antigen‐A3 through YAP. In conclusion, sitagliptin may have a potential inhibitory effect on GC by AMPK/YAP/melanoma‐associated antigen‐A3 pathway.

## Graphical abstract

Sitagliptin inhibits the proliferation and clonality of gastric cancer cells, which was accompanied by increased levels of adenosine 5′‐monophosphate‐activated protein kinase phosphorylation and Yes‐associated protein (YAP) phosphorylation expression and decreased YAP nuclear localization. In addition, sitagliptin reduces melanoma‐associated antigen‐A3 expression via the YAP pathway, which may improve the prognosis of gastric cancer.

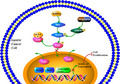

## 1 INTRODUCTION

Diabetes is a chronic metabolic disease with an increasing prevalence. An estimated 642 million people all over the world aged between 20 and 79 years old will develop in diabetes by 2040.^1^ Recently, growing epidemiological evidence has shown an increased risk of gastric cancer (GC) among diabetic patients. One potential reason may be that these two diseases involve common risk factors, including hyperglycemia, hyperinsulinemia, and *Helicobacter pylori* infection.^2^ Therefore, further exploring the antitumor mechanism of hypoglycemic agents is of great significance for diabetic patients with GC.

As a new oral hypoglycemic agent, sitagliptin has been widely used in many countries. Sitagliptin, as a dipeptidyl peptidase‐IV (DPP4) inhibitor, could reduce glucagon levels and increase insulin levels by promoting the secretion of intestinal hormones.^3^ Previous researches have shown that sitagliptin can reduce the risk of multiple tumors including breast,^4^ kidney,^5^ and colon cancer,^6^ as well as improve the prognosis. However, there are few reports on the effects of sitagliptin on GC. Therefore, we aimed to provide new options for the treatment of GC by exploring the molecular mechanism of sitagliptin in regulating GC.

Genome sequencing results show that there are frequent mutations in signaling pathways in GC.^7^ The Hippo pathway, as a frequently mutated pathway in GC, is an essential process in multiple steps affecting adverse outcomes of GC, and is a new approach investigation worthy of further development.^8^ First identified in *Drosophila*, the Hippo signaling pathway is a cell signal transduction pathway. Later, its homologous proteins were found in mammals. It is mainly composed of a series of tandem kinases, which plays an important role in tissue homeostasis and tumor development.^9^ The main mechanism of Hippo signaling hinges on whether the most downstream nuclear cotransfer factor Yes‐associated protein (YAP) can enter into the nuclear and activate its downstream transcription factors.^10^ Genetic studies have established that the large tumor suppressor homolog (LATS) kinase is the upstream kinase of YAP and could inhibit its activity by phosphorylating YAP. Un‐phosphorylated YAP can enter the nucleus to play a role in promoting cancer, while phosphorylated YAP binds to specific proteins in the cytoplasm, so it is inactive to stimulate gene expression.^9^, ^11^ Abnormal nuclear YAP overexpression is found in various tumors such as lung,^12^ gastric,^13^ and pancreatic ductal adenocarcinoma,^14^ etc Moreover, the upregulation of nuclear YAP promotes tumorigenesis and is closely related to poor prognosis.^12^, ^14^ The inactivation regulation of tumor cell growth has been confirmed to be a feature of tumorigenesis. Thus, the highly conserved Hippo signaling pathway is closely related to tumorigenesis.

Previous studies of the Hippo pathway have demonstrated that several molecules can affect and alter the activity of YAP. For example, adenosine 5′‐monophosphate‐activated protein kinase (AMPK) can phosphorylate YAP in a LATS‐dependent or LATS‐independent manner to inhibit YAP activity,^15^ and the regulation of YAP by AMPK may be related to tumor cell proliferation and tumor resistance.^16^ More importantly, it has been reported that sitagliptin can affect inflammation, autophagy, and metabolism by regulating AMPK.^3^, ^17^ These mechanisms suggest that AMPK/YAP may represent a novel target for sitagliptin to inhibit tumor growth.

It is known that tumor antigens can induce a strong specific immune response in the body, which can provide new ideas for antitumor treatment.^18^ Among many tumor antigens, more and more tumor‐testis antigens have attracted the attention of researchers. Such antigens include sal‐like gene family, melanoma‐associated antigen gene family, and synovial sarcoma X breakpoint gene family,^18^, ^19^ etc. Melanoma‐associated antigen‐A3 (MAGE‐A3) belongs to the melanoma antigen family and has the characteristics of being expressed in a variety of tumor tissues, but hardly expressed in normal tissues except testicular and trophoblast cells. Moreover, MAGE‐A3 can bind to human leukocyte antigen molecules and play a specific killing effect on tumor cells.^20^ Melanoma‐associated antigen‐A3 is over‐expressed in many malignant tumors (eg, colorectal and liver cancer) and has a high degree of specificity, especially in GC, with the positive expression rate up to 40%.^21^ The current researches on MAGE‐A3 indicate that MAGE‐A3 can promote tumor cell proliferation and affect tumor prognosis by regulating multiple apoptotic proteins.^22^ Thus, we hypothesized that YAP/MAGE‐A3 may be a new target for sitagliptin.

This study demonstrates that sitagliptin exerts an anticancer function through AMPK/YAP/MAGE‐A3 signaling axis and provides new options for the treatment of GC.

## 2 MATERIALS AND METHODS

### 2.1 Tissue specimens

Tumor tissues were obtained from patients who were diagnosed with GC and undergoed surgery at Shengjing Hospital affiliated to China Medical University from 2012 to 2013. The research was approved by the Ethics Committee of the First Affiliated Hospital of China Medical University.

### 2.2 Cell culture

The human GC cell lines, human gastric adenocarcinoma cell line (AGS), human gastric cancer cell line (HGC‐27), and MKN45, were acquired from the Department of Biochemistry, China Medical University. AGS cells were cultured using DMEM‐F12 medium (GE Healthcare Life Science HyClone Laboratories). HGC‐27 and MKN45 cells were cultured using 1640 medium (GE Healthcare Life Science HyClone Laboratories). The medium contained 10% of FBS (Biological Industries), 1% of antibodies, and 1% of glutamine. The culture conditions were maintained at 37℃, 5% of CO_2_ in a humid environment.

### 2.3 Cell viability assay

This experiments were evaluated in accordance with the cell counting kit‐8 (CCK8) reagent instructions. GC cells (AGS, HGC‐27, and MKN45 cells) were cultured in 96‐well plates at appropriate densities. After the synchronization treatment, GC cells were treated with 0, 0.5, 1, 1.5, 2, 2.5, 3, 3.5, 4, and 4.5 mmol/L of sitagliptin for 24 hours. The transfected cells were redigested after transfection for 48 hours and incubated for the designated time points (0, 24, 48, 72, and 96 hours). The medium was changed by adding 100 μL of serum‐free medium containing 10 μL of CCK8 working solution. After another incubation for 2 hours in the dark, shake at room temperature for 3 seconds. The multiscan spectrophotometer (model: Infinite M200; Tecan Trading) measured the absorbance of each well at 450 nm to evaluate the degree of cell proliferation.

### 2.4 Colony formation assay

Gastric cancer cells (AGS, HGC‐27, and MKN45 cells) in logarithmic growth phase were digested, resuspended, and evenly spread in 6‐well plates (500 cells/well). After the cells adhered to the dish, the corresponding treatment factors were continued for 7‐10 days. After the colonies were formed, the 6‐well plate was taken out and washed. Subsequently, they were respectively treated with methanol and crystal violet for 30 minutes to complete the dyeing. After drying at room temperature, the numbers of clones comprising more than 50 cells were counted under light microscopy.

### 2.5 Immunofluorescence

The GC cells were digested and uniformly cultured on the coverslips at a suitable density, and treated with sitagliptin for 24 hours after the cells had fully adhered. After completing the scheduled processing, the cells in each well were fixed in cold 4% of paraformaldehyde. In order to increase the permeability of the cell membrane to the antibody, pretreated the cells with 0.3% of Triton‐X100 for another 15 minutes, and then, soaked the cells in a 5% of bovine serum albumin. Subsequently, the coverslips were completely covered with a 1:200 dilution of YAP antibody and incubated overnight in a 4°C wet box. The next day, the cells were incubated with fluorescein isothiocyanate‐conjugated secondary antibodies (Molecular Probes) for 60 minutes. Finally, 4′,6‐diamidino‐2‐phenylindole was counterstained for 5 minutes in the dark. Examined the changes in the expression of fluorescent protein positions with a fluorescence microscope (Olympus Corp.).

### 2.6 RNA extraction and analysis

Total RNA was extracted according to TRIzol Reagent (Thermo Fisher Scientific (China) Inc) extraction protocol. cDNA was used as a template to detect the level of the mRNA expression of related genes (YAP, CTGF, CYR61, and MAGE‐A3) by real‐time polymerase chain reaction (PCR) (Promega Corporation). We used 18S as an internal reference. The primers were detailed in Table 1. Using 2^−ΔΔCt^ to calculate relative expression differences between genes.

**TABLE 1:** Primes used for human cell lines

Gene	Forward	Reverse
18S	GCAGAATCCACGCCAGTACAAGAT	TCTTCTTCAGTCGCTCCAGGTCTT
CTGF	CCAATGACAACGCCTCCTG	TGGTGCAGCCAGAAAGCTC
CYR61	AGCCTCGCATCCTATACAACC	TTCTTTCACAAGGCGGCACTC
MAGE‐A3	AAGCCGGCCCAGCRCGGT	GCTGGGCAATGGAGACCCAC

### 2.7 Western blot

The protein was extracted first. The total protein was extracted by scraping the cells with an appropriate amount of cell lysate, then, sonicating the cells for full lysis, and finally, centrifuging to obtain the total protein. To obtain nuclear plasma‐separated protein, a nuclear plasma‐separation kit (Beyotime Biotechnology) was used, which was performed in accordance with the steps of the reagent vendor. The concentration of the protein was measured using bicinchoninic acid (BCA) protein assay kit (Beijing Solarbio Science & Technology Co., Ltd.). After separating thirty microgram proteins of different molecular weights by 4%‐12% of Sodium dodecyl sulfate polyacrylamide gel electrophoresis (SDS‐PAGE) (GenScript USA Inc), the proteins were transferred to a 0.45 μm of polyvinylidene fluoride (PVDF) membrane. After blocking in 5% of bovine serum albumin for 2 hours at room temperature, the membranes were placed in the corresponding diluted primary antibody (anti‐p‐LATS Ser909, anti‐LATS; anti‐p‐YAP Ser127; anti‐YAP; anti‐MAGE‐A3; anti‐glyceraldehyde‐3‐phosphate dehydrogenase (GAPDH); anti Histon‐H3), and incubated overnight on a shaker at 4℃. The membranes were removed from the antibody cassette, and incubated with the corresponding horseradish peroxidase (HRP) secondary antibody for 2 hours on a shaker. The electrochemiluminescence (ECL) kit (China, Wanleibio Co., Ltd.) was used to detect the band of the antigen‐antibody binding region, and the expression of proteins with different molecular weights was compared by quantitative analysis of optical density values.

### 2.8 Transfection

Yes‐associated protein 5SA (YAP phosphorylation site mutant plasmid), YAP control (Xiamen University, China), siRNA YAP (Sigma‐Aldrich, Inc), siRNA MAGE‐A3 (Sigma‐Aldrich, Inc), and siRNA control were transfected into AGS, HGC‐27, and MKN45 cells using Lipofectamine 3000 (Thermo Fisher Scientific (China) Inc). The plasmid, siRNA, and Lipofectamine 3000 were gently dissolved in Opti‐MEM (Thermo Fisher Scientific (China) Inc), and mixed at room temperature. Then, the mixed solution was dropped into the cell culture dish after 15 minutes, and finally, returned them to the incubator. After 48 or 72 hours of transfection, make appropriate arrangements according to the needs of subsequent experiments.

### 2.9 Immunohistochemistry and quantitation

In order to investigate the connection between MAGE‐A3 and GC prognosis, we tested the positive rate of MAGE‐A3 in 66 GC patients by immunohistochemistry, and analyzed the connection between MAGE‐A3 and pathological parameters. The positive ratio of the tumor cells was evaluated according to the following scoring criteria: <25%, (a); 25%‐50%, (b); 50%‐75%, (c); and >75%, (d). The staining intensity was judged according to three categories: weak, (a); mild, (b); and strong, (c). The results were divided into two groups according to the staining index (staining intensity score × positive rate score): 0‐4 points constituted the low expression group; 5‐12 points were used to classify the high expression group.

### 2.10 In silico analysis

The mRNA expression profile (GSE54129) was obtained from the gene expression omnibus (GEO) database (http://www.ncbi.nlm.nih.gov/geo/). We used standardized data for the analysis. The Kaplan‐Meier plotter (http://kmplot.com/analysis/) was used to evaluate the relationship between MAGE‐A3 and GC survival.^23^ The data were processed by GraphPad Prism v7.0.4 (GraphPad Software Inc).

### 2.11 Statistical analysis

The data presented were repeated at least three times independently, and were expressed as the mean ± SD. Differences between groups were analyzed using *t* test, and a threshold of *P* < .05 was considered statistically significant.

## 3 RESULTS

### 3.1 Sitagliptin inhibits GC cells proliferation

**FIGURE 1:**
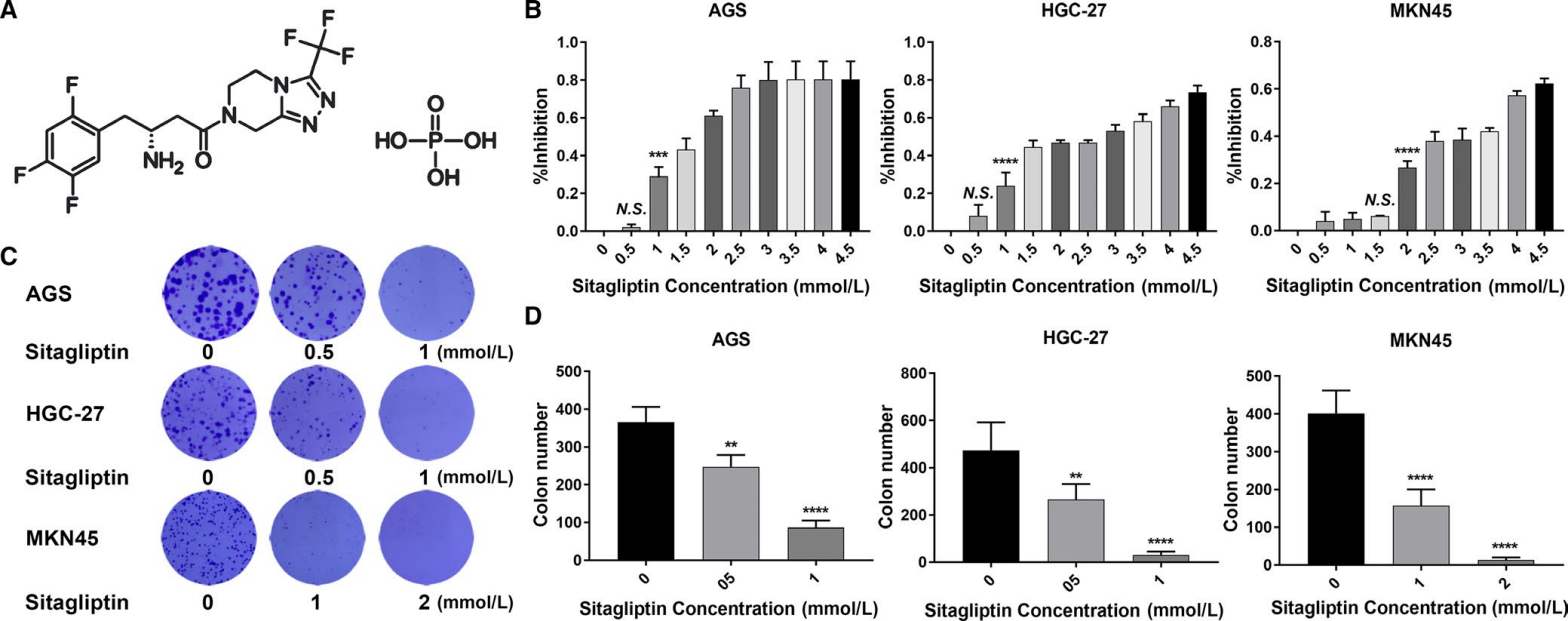
Sitagliptin inhibits gastric cancer cells proliferation. A, The structure of sitagliptin; (B) The CCK8 assay was used to analyze the inhibition rate after AGS, HGC‐27, and MKN45 cells treated with 0, 0.5, 1, 1.5, 2, 2.5, 3, 3.5, 4, 4.5 mmol/L sitagliptin for 24 h; (C) Colony formation assay was performed to assess the long‐term effect of sitagliptin on AGS, HGC‐27, and MKN45 cells; (D) statistically evaluated the difference in clone numbers between treatment groups. NS represented not significant; **P < .01; ***P < .005; ****P < .001

We first determined whether sitagliptin (structure shown as Figure 1A) could inhibit GC cell proliferation. To this end, three human‐derived GC cells (AGS, HGC‐27, and MKN45 cell lines) were stimulated with 0, 0.5, 1, 1.5, 2, 2.5, 3, 3.5, 4, and 4.5 mmol/L of sitagliptin for 24 hours, and then, the cell viability was measured. The inhibitory effect of sitagliptin on the GC cell lines increased with the increase of sitagliptin concentration (Figure 1B). To further demonstrate the relationship between decreased cell viability and sitagliptin, we tested the ability of sitagliptin on the colony formation ability of the GC cell lines. Figure 1C,D showed that sitagliptin treatment reduced the number of colonies. The above results indicated that sitagliptin inhibited the GC cells proliferation.

### 3.2 Sitagliptin induces YAP phosphorylation in GC cells

**FIGURE 2:**
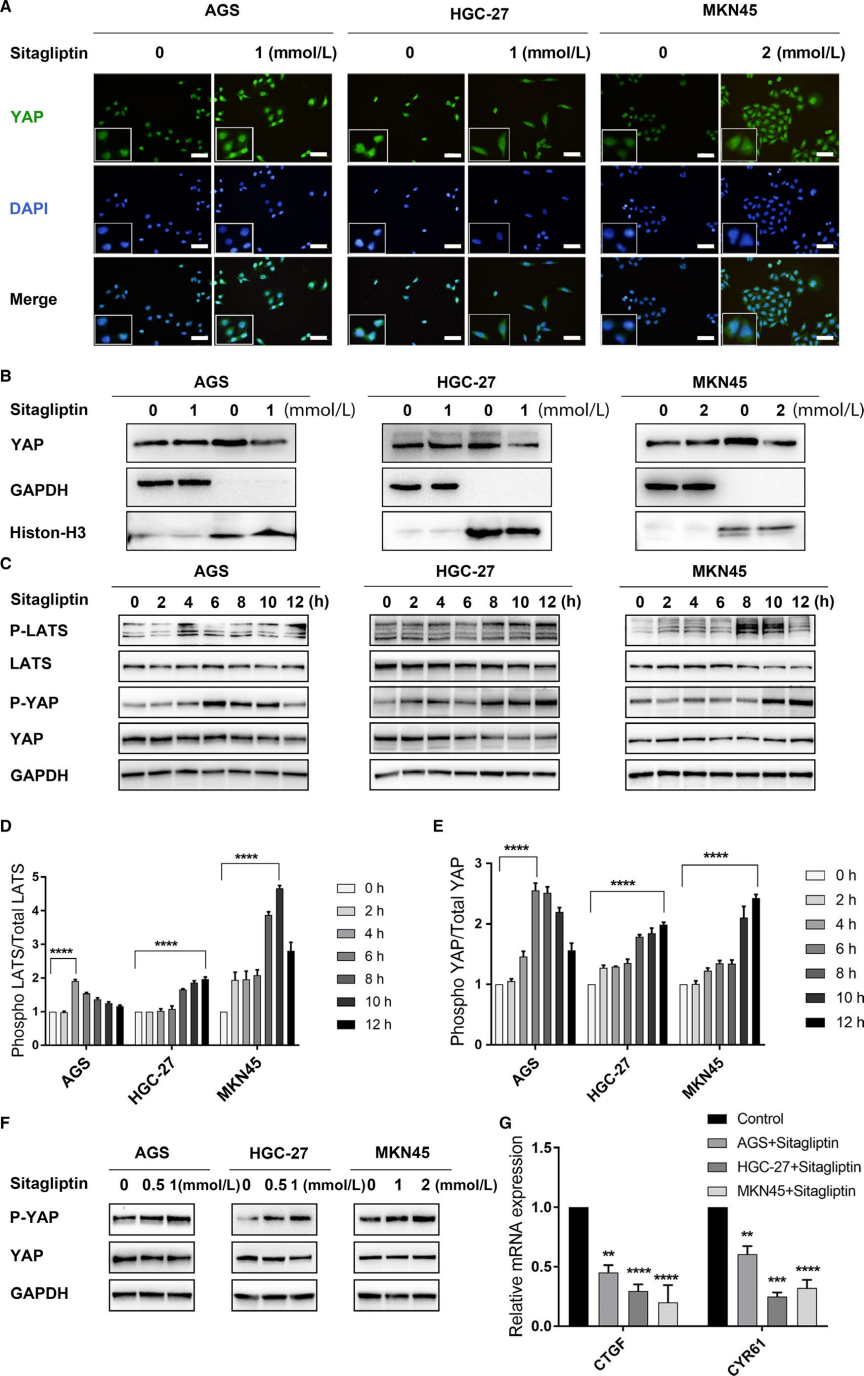
Sitagliptin induces Yes‐associated protein (YAP) phosphorylation in gastric cancer cells. A, Immunofluorescence was used to examine the distribution of YAP in sitagliptin‐treated gastric cancer cells. Green represented YAP, blue represented the nucleus (magnification 400×, scale bar 20 µm); (B) the nuclear and cytoplasmic protein extraction technology was used to clarify the effect of sitagliptin on the expression of YAP in the nucleus. The protein expression of YAP was examined using Western blot. GAPDH was used as cytoplasmic reference, while Histon‐H3 was used as nuclear reference; (C) Western blot analysis was used to evaluate p‐large tumor suppressor homolog (LATS) (Ser 909), LATS, p‐YAP (Ser 127), and YAP protein expression with GAPDH as control; (D, E) the peak level of phosphorylation normalized by total protein level compared with control group. Each cell line had a peak of phosphorylation; (F) sitagliptin was added to the gastric cancer (GC) cells, treated with AGS for 6 h, and HGC‐27 and MKN45 for 12 h. YAP and p‐YAP were examined using Western blot; (G) the effects of sitagliptin on the mRNA expression of connective tissue growth factor (CTGF) and cysteine‐rich angiogenic inducer 61 (CYR61). **P < .01; ***P < .005; ****P < .001

The Hippo pathway is crucial in multiple steps affecting poor prognosis of GC, especially regarding nuclear YAP over‐expression.^8^, ^13^ Considering that YAP is a nuclear cotranscription factor, its ability to enter the nucleus is of great significance for its cancer‐promoting function. Therefore, we first used immunofluorescence to detect changes in the nuclear localization of YAP after sitagliptin treatment. Blue expression represented the nucleus, green expression represented YAP. Figure 2A showed that after the sitagliptin treatment, the green fluorescence in the nucleus reduced significantly. To further clarify the effect of sitagliptin on nuclear YAP expression, nuclear, and cytoplasmic protein extraction technology was used. As we expected, the expression of YAP in the nucleus decreased significantly as a result of sitagliptin treatment (Figure 2B). We all know that only un‐phosphorylated YAP can enter the nucleus and bind to downstream factors, while phosphorylated YAP can only hold in the cytoplasm and wait for degradation.^9^, ^11^ Therefore, the expression of p‐YAP (Ser127), YAP and their upstream regulatory kinases p‐LATS (Ser909), and LATS were assessed using Western blot. GC cells were treated with effective concentration of sitagliptin for 0, 2, 4, 6, 8, 10, and 12 hours, and the expression of related proteins was detected. Figure 2C revealed that sitagliptin upregulated the expression of p‐LATS (Ser909) and p‐YAP (Ser127) in all cell lines. We analyzed the peak level of phosphorylation normalized by total protein level compared with control group. Each cell line had a peak of phosphorylation (Figure 2D,E). Next, different concentrations of sitagliptin were added to the GC cells (AGS and HGC‐27 cells: 0.5 and1 mmol/L; MKN45 cells: 1 and 2 mmol/L) at the time point exhibiting the highest level of p‐YAP (Ser127) expression (AGS treated for 6 hours, HGC‐27 and MKN45 for 12 hours). The results revealed that sitagliptin treatment increased the level of p‐YAP (Ser127) expression in a dose‐dependent manner (Figure 2F). Connective tissue growth factor (CTGF) and cysteine‐rich angiogenic inducer 61 (CYR61) are two known YAP effector factors. Moreover, the level of expression of these factors can reflect the nuclear localization of YAP to some extent.^24^ Thus, the level of CTGF and CYR61 expression was examined in sitagliptin‐treated GC cells using real‐time PCR. The results showed that CTGF and CYR61 were downregulated by sitagliptin at the mRNA level (Figure 2G). In conclusion, these findings suggested that sitagliptin inhibited YAP entering into the nucleus through YAP phosphorylation and reduced YAP expression in the nucleus of GC cells.

### 3.3 AMPK inhibition reverses sitagliptin‐induced YAP phosphorylation in GC cells

**FIGURE 3:**
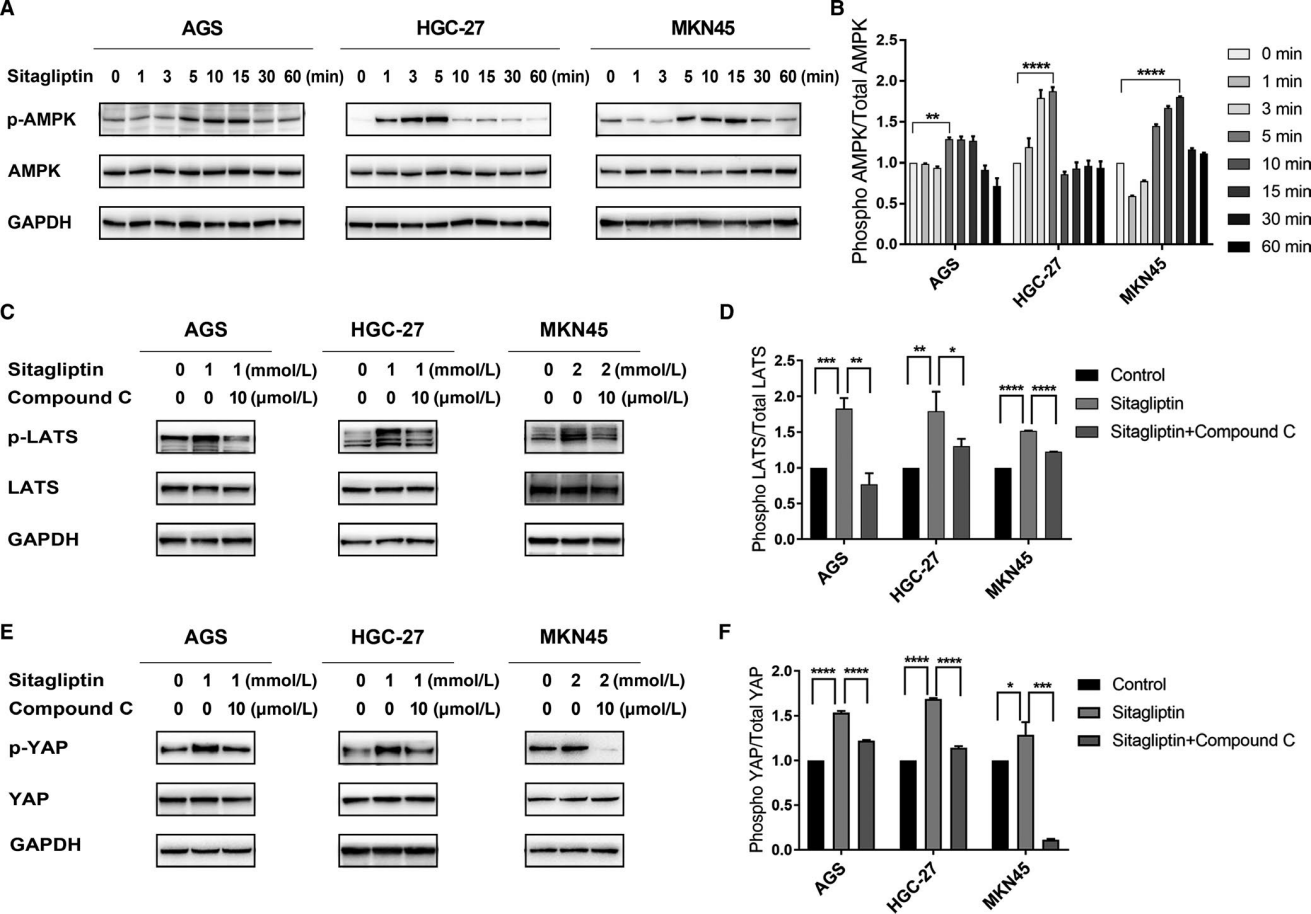
AMP‐activated protein kinase (AMPK) inhibition reverses sitagliptin‐induced Yes‐associated protein (YAP) phosphorylation in gastric cancer (GC) cells. (A) Western blot analysis was carried out to evaluate the expression of p‐AMPK and AMPK, with GAPDH as control; (B) the peak level of phosphorylation normalized by total protein level compared with control group; (C) after pretreated with or without 10 µm Compound C, GC cells were treated with sitagliptin (AGS for 4 h, HGC‐27 for 12 h and MKN45 for 10 h). Western blot analysis was used to evaluate p‐LATS (Ser 909) and LATS protein expression. GAPDH was used as control; (D) the peak level of phosphorylation normalized by total protein level compared with control group; (E) after pretreated with or without 10 µm Compound C, GC cells were treated with sitagliptin (AGS for 6 h, HGC‐27 for 12 h and MKN45 for 12 h). Western blot analysis was used to evaluate p‐YAP (Ser 127) and YAP protein expression; (F) the peak level of phosphorylation normalized by total protein level compared with control group. *P < .05; **P < .01; ***P < .005; ****P < .001

It has been previously confirmed that sitagliptin can activate AMPK.^3^, ^17^ Activated AMPK can inhibit YAP activity by directly or indirectly increasing the level of YAP phosphorylation.^15^ Based on these studies, we hypothesized that the inhibitive role of sitagliptin on GC cells and YAP was regulated by AMPK. To verify this hypothesis, the effect of sitagliptin on the level of AMPK phosphorylation (p‐AMPK) was examined. GC cells were stimulated by an effective dose of sitagliptin (1 mmol/L for AGS and HGC‐27; 2 mmol/L for MKN45) for 0, 1, 3, 5, 10, 15, 30, and 60 minutes. The level of p‐AMPK was significantly increased within 15 minutes (Figure 3A,B). To confirm that the inhibitive role of sitagliptin on the nuclear expression of YAP was regulated by AMPK, 10 µm of the AMPK inhibitor, Compound C, was used to pretreat the cells for 2 hours to inhibit AMPK activity. As expected, pretreatment with Compound C blocked the expression of p‐LATS and p‐YAP induced by sitagliptin (Figure 3C‐F). In summary, all the results indicated that AMPK regulated the activation of YAP phosphorylation by sitagliptin in GC cells.

### 3.4 Sitagliptin inhibits MAGE‐A3 expression in GC cells

**FIGURE 4:**
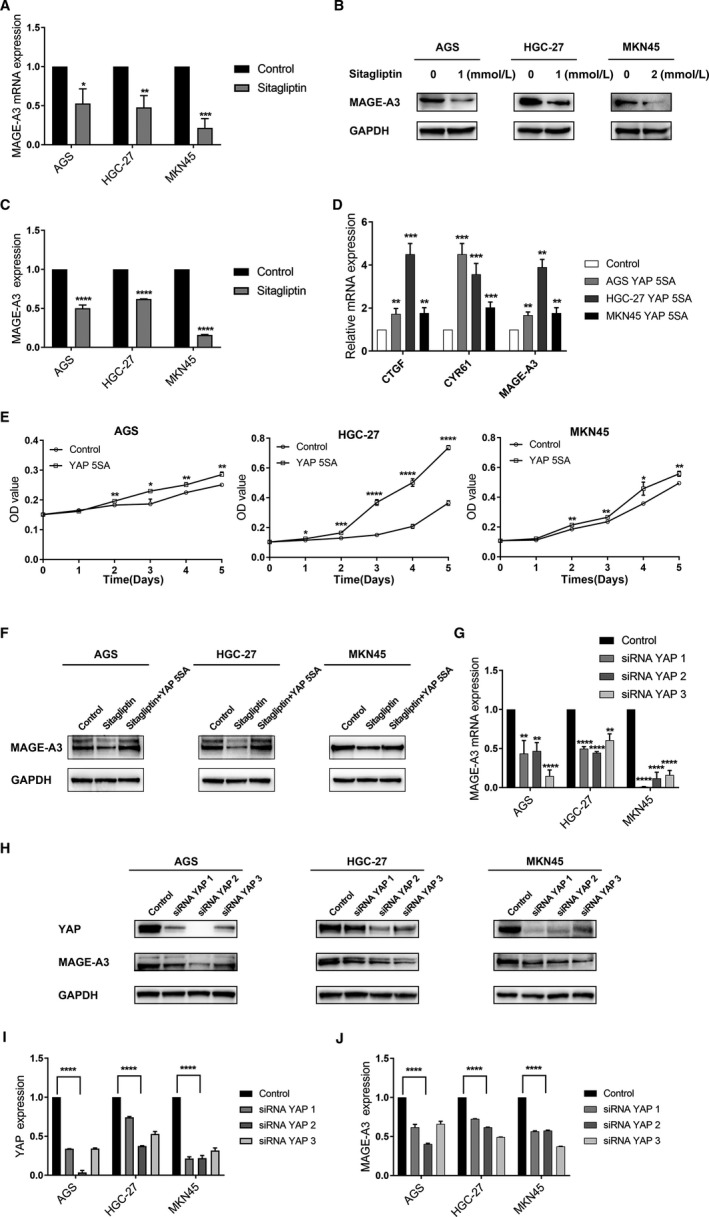
Sitagliptin inhibits melanoma‐associated antigen‐A3 (MAGE‐A3) expression in gastric cancer cells. A and B, The mRNA and protein expression of MAGE‐A3 in gastric cancer (GC) cells after sitagliptin treatment; (C) Statistical evaluation of differences in MAGE‐A3 protein levels; (D) after transfection with Yes‐associated protein (YAP) 5SA or YAP control, the mRNA expression of connective tissue growth factor (CTGF), cysteine‐rich angiogenic inducer 61 (CYR61), and MAGE‐A3 were examined using real‐time PCR; (E) after transfection with YAP 5SA or YAP control, cell viability was tested by CCK8 assay; (F) after transfection with YAP 5SA or YAP control, cells were treated with sitagliptin (1 mmol/L for AGS and HGC‐27; 2 mmol/L for MKN45). MAGE‐A3 protein expression were examined by Western blot, with GAPDH as control; (G) after transfection with siRNA YAP or siRNA control, MAGE‐A3 mRNA expression was confirmed using real‐time PCR; (H) the expression of YAP and MAGE‐A3 were examined to confirm that knocking down YAP did reduce the level of MAGE‐A3; (I, J) statistical evaluation of differences in YAP and MAGE‐A3 protein levels. *P < .05; **P < .01; ***P < .005; ****P < .001

It is well known that YAP is a cotranscription factor, which cannot work directly, so we need to find the effect factor sitagliptin plays. The tumor/testicular antigen family is specifically and stably expressed only in tumors, which has been an area of keen interest since its discovery.^20^ Studies have shown that BCL2‐associated athanogene 1, melanoma‐associated antigen‐A1, MAGE‐A3, melanoma‐associated antigen‐C2, SSX family member 2, and SSX family member 4 genes are several representative genes that are specifically and highly expressed in stomach cancer.^19^, ^25^, ^26^ We downloaded the relevant gene mRNA expression in stomach cancer using the GEO database (GSE54129) and tested the correlation between these genes and YAP with a correlation analysis. Melanoma‐associated antigen‐A3 was found to have the highest correlation with YAP in GC (Figure S1). Since MAGE‐A3 is specifically overexpressed in GC and is closely related to GC cells proliferation,^22^ then, if sitagliptin can exert anticancer effects by downregulating MAGE‐A3, it will provide a new therapeutic target for GC. To verify this hypothesis, GC cells were treated with sitagliptin (1 mmol/L for AGS and HGC‐27; 2 mmol/L for MKN45) for 24 hours and the expression of MAGE‐A3 was detected. As expected, MAGE‐A3 expression was significantly decreased at both the mRNA and protein level (Figure 4A‐C). Although sitagliptin can reduce MAGE‐A3 expression, it is still necessary to prove that sitagliptin's effect on MAGE‐A3 is related to YAP. It has been reported that YAP 5SA mutations promote YAP constitutive nuclear localization and transcriptional activation by inhibiting YAP phosphorylation.^27^ As shown in Figure 4D, after transfection of YAP 5SA plasmids, the expression of CTGF and CYR61 increased compared to the control group, while the mRNA expression of MAGE‐A3 also increased. In addition, we can see that after transfection, the proliferation of stomach cancer cells significantly accelerated (Figure 4E). Most importantly, we detected the effect of sitagliptin on MAGE‐A3 expression after the transfection of YAP 5SA. As shown in Figure 4F, sitagliptin can reduce MAGE‐A3 expression, but after transfecting YAP 5SA, the plasmids blocked the expression of MAGE‐A3 induced by sitagliptin. Moreover, the level of total YAP can also reflect the regulation of MAGE‐A3 by sitagliptin at a certain level, so a knock down of YAP was performed. The results showed that the YAP knock down did reduce the level of MAGE‐A3 at both the transcriptional and protein levels (Figure 4G‐J). Taken together, these results indicated that sitagliptin regulated MAGE‐A3 via YAP, thereby exerting an anticancer effect.

### 3.5 Correlation between MAGE‐A3 expression and GC survival

**FIGURE 5:**
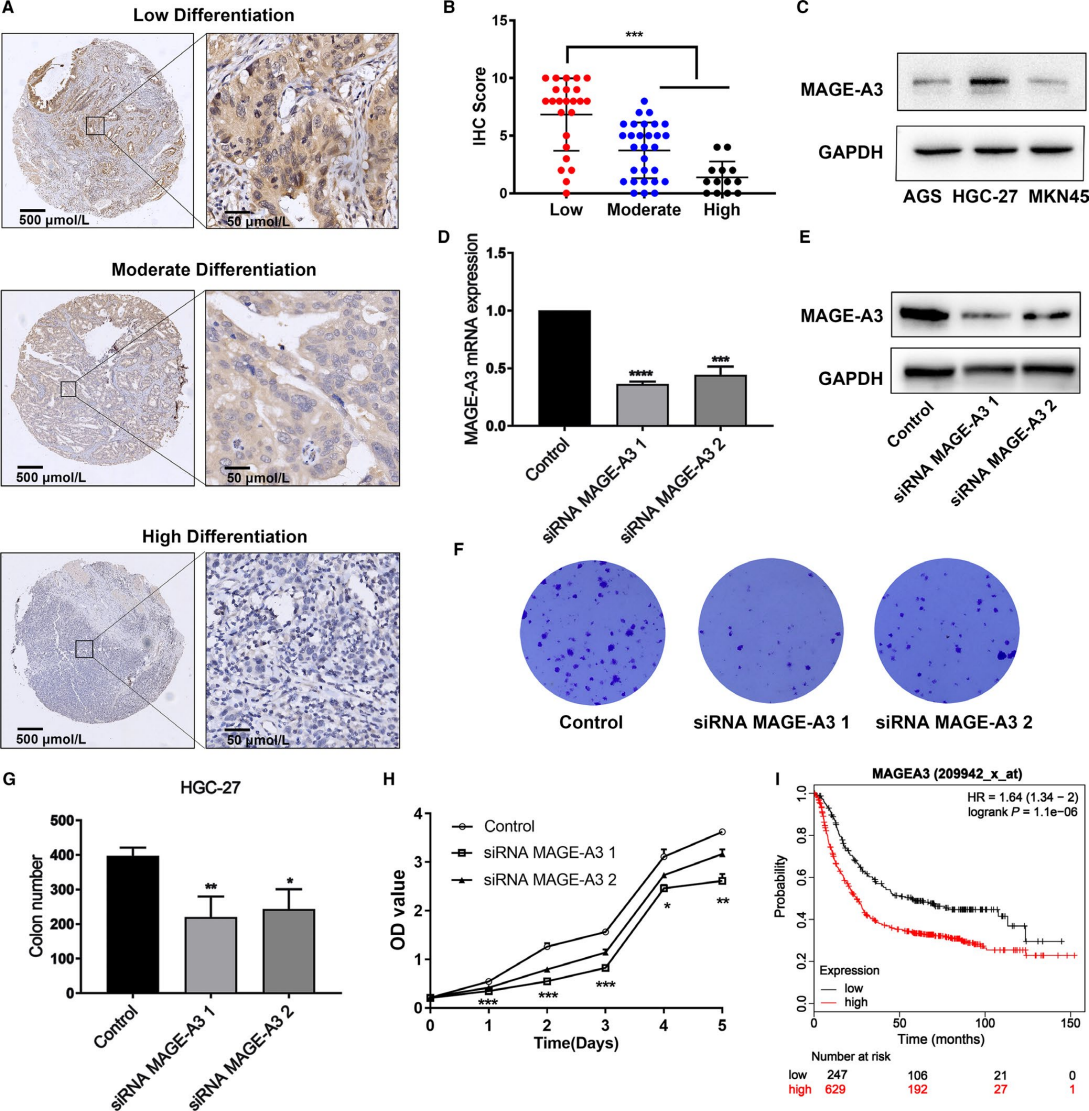
Correlation between melanoma‐associated antigen‐A3 (MAGE‐A3) expression and gastric cancer survival. A, MAGE‐A3 expression was determined in gastric cancer (GC) by immunohistochemistry (IHC) (low differentiation; moderate differentiation; high differentiation). The data were shown at different magnification (4 × and 40×); (B) IHC expression of MAGE‐A3 quantified by staining score (0‐12) in GC tissues; (C) Western blot analysis was used to evaluate MAGE‐A3 expression in AGS, HGC‐27, and MKN45 cells, with GAPDH as control; (D, E) knocking down MAGE‐A3 expression in HGC‐27 cells by targeted siRNA transfection. The mRNA and protein expression of MAGE‐A3 were examined; (F) colony formation assay was performed to assess the long‐term effect of siRNA MAGE‐A3 on HGC‐27 cells; (G) statistically evaluated the difference in clone numbers between treatment groups; (H) CCK8 assay was used to assess the cell viabilities of HGC‐27 cells treated with siRNA MAGE‐A3 or siRNA control for different days; (I) the Kaplan‐Meier plotter website data were used for a survival analysis. *P < .05; **P < .01; ***P < .005; ****P < .001

In order to explore the correlation between MAGE‐A3 and GC prognosis, immunohistochemical methods were used to analyze the pathological specimens of 66 patients with GC. Table 2 indicated that MAGE‐A3 was associated with cancer differentiation and lymph node metastasis, but was unassociated with age, tumor size, Her2, or Lauren. Since MAGE‐A3 is closely related to differentiation, there is a close relationship between differentiation and tumor prognosis. Therefore, we created high, moderate, and low differentiation groups, to show the specific expression of MAGE‐A3 in stomach cancer. In Figure 5A,B, MAGE‐A3 was highly significantly expressed in low‐differentiated stomach cancer compared with high‐differentiated. To clarify the effect of MAGE‐A3 on proliferation, we selected non‐differentiated GC cells (HGC‐27), which exhibited a relatively high level of MAGE‐A3 expression, for all subsequent experiments (Figure 5C). The expression of MAGE‐A3 in HGC‐27 cells was knocked down with targeted siRNA transfection (Figure 5D,E). After confirming the successful transfection, both the control and knockdown groups of HGC‐27 cells were used to detect the cell proliferation and colony formation ability. The experimental data showed that the cell proliferation and colony forming ability were significantly decreased following a knockdown of MAGE‐A3 (Figure 5F‐H). To further verify whether MAGE‐A3 was indeed related to prognosis, we used the Kaplan‐Meier plotter website data for a survival analysis, and found that GC patients with a high MAGE‐A3‐expressing did not live as long as the low expression groups (Figure 5I). In conclusion, these results indicated that MAGE‐A3 promoted GC cell proliferation, which resulted in a poor prognosis in GC patients.

**TABLE 2:** Correlations between expression of MAGE‐A3 protein and the clinicopathological features of patients

Characteristic	Case (n)	MAGE‐A3 expression		*P* value	*r* value
		Positive	Negative		
Age				.534	−0.078
≤60	27	18 (66.67%)	9 (33.33%)		
>60	39	23 (58.97%)	16 (41.03%)		
Lymph node metastasis				.012*	0.309
No	22	9 (40.91%)	13 (59.09%)		
Yes	44	32 (72.73%)	12 (27.27%)		
Tumor size				.803	−0.031
<5 cm	33	21 (63.63%)	12 (36.37%)		
≥5 cm	33	20 (60.61%)	13 (39.39%)		
Differentiation				.001****	0.39
Low	24	19 (79.17%)	5 (20.83%)		
Moderate	29	17 (58.62%)	12 (41.38%)		
High	13	2 (15.38%)	11 (84.61%)		
Her2				.213	0.181
Negative	26	16 (61.54%)	10 (38.46%)		
Positive	23	18 (78.26%)	5 (21.74%)		
Unknown	17				
Lauren					
Intestinal type	16	13 (81.250%)	3 (18.75%)	.065	−0.298
Diffuse type	23	12 (52.17%)	11 (47.83%)		
Unknown	27				

Abbreviation: MAGE‐A3, melanoma‐associated antigen‐A3. *P < .05; ****P < .001

## 4 DISCUSSION

With changes in lifestyle and life rhythm, the prevalence of diabetes has increased annually. Metabolic disorders and chronic inflammation caused by diabetes promote the development of GC.^28^ Exploring the antitumor mechanism of hypoglycemic agents can both provide new therapeutic targets for tumor treatment, and provide more reasonable treatment options for patients with diabetes and tumors.

Sitagliptin is a widely used oral hypoglycemic agent. Moreover, studies have reported that sitagliptin inhibits the development of various tumors both in vitro and in vivo.^5^, ^6^ Till now, research regarding the inhibition of cancers by sitagliptin has primarily focused on the analysis of clinical data, and knowledge of its molecular mechanism remains limited. The currently known mechanism of sitagliptin‐mediated tumor suppression mainly involves the function of sitagliptin as a DPP4 inhibitor, which can inhibit epithelial‐mesenchymal transition and tumor metastasis by inhibiting DPP4 expression, thereby improving tumor immunity and immunotherapy.^29^ In addition, sitagliptin can regulate the nuclear factor kappa‐B (NF‐κB) pathway and exert direct anti‐inflammatory effects.^30^ In this study, our results demonstrated that sitagliptin inhibited the proliferation and colony‐forming ability of GC cells. We then explored the potential mechanisms of sitagliptin inhibiting GC cell proliferation.

AMP‐activated protein kinase is a eukaryotic energy receptor that is important for maintaining the balance of energy metabolism in cells.^31^ In fact, as a potential target for tumor therapy, AMPK can benefit a variety of malignancies by interfering with tumor cell metabolism and cancer proliferation.^32^ Our results indicated that sitagliptin activated AMPK and inhibited the proliferation and clonality of GC cells, suggesting that AMPK activation can antagonize the proliferation of GC. Previous reports have shown that in the case of energy stress, AMPK activation can directly or indirectly phosphorylate YAP, thereby inhibiting the entry of YAP into the nucleus.^15^, ^33^ As a hypoglycemic agent, sitagliptin easily causes changes in energy metabolism. Thus, we further tested whether AMPK also inhibited YAP in sitagliptin‐treated GC cells. We found that sitagliptin‐induced YAP cytoplasmic retention by promoting the phosphorylation of the YAP and prevented the nuclear activation required for its cancer‐promoting function. Inhibition of AMPK reversed the inhibitory effect of sitagliptin on YAP. This indicated that AMPK/YAP was a potential therapeutic target for GC.

As a proto‐oncogene, YAP is involved in multiple pathophysiological processes in disease development (eg, tumor proliferation, invasion and metastasis, dry maintenance, energy metabolism, and cell resistance) and plays important roles in maintaining homeostasis.^9^, ^11^, ^12^ It is well‐established that some drugs (eg, verteporfin) can exert anticancer effects by inhibiting the binding of YAP to transcription enhancer factors.^34^ We found that sitagliptin could exert an anticancer effect by inducing the phosphorylation of YAP, which caused YAP unable to accumulate in the nucleus. And the induction of this phosphate was related to the phosphorylation of LATS. In investigating this process, we focused on MAGE‐A3, a member of tumor‐testis antigens family. MAGE‐A3 is an emerging prognostic factor for GC and is likely a marker for the early diagnosis and micrometastasis identification of GC.^21^, ^22^ In this study, we also verified that MAGE‐A3 was closely related with poor prognosis of GC. Knocking down MAGE‐A3 significantly inhibited the proliferation of GC cell lines. Sitagliptin was effective to inhibite the expression of MAGE‐A3. This inhibition was closely related with YAP expression. Therefore, we hypothesized that sitagliptin inhibited GC cell proliferation via YAP/MAGE‐A3 signaling. Since MAGE‐A3 is hardly expressed in normal tissues, this target molecule provides an ideal site for GC targeted therapy.^25^ Since there are many complex regulatory networks between various signaling pathways, our results may not cover the mechanisms of sitagliptin inhibition of tumors, but complement other findings. Further research is required regarding the specific mechanisms of action and safety issues associated with sitagliptin in GC.

**FIGURE 6:**
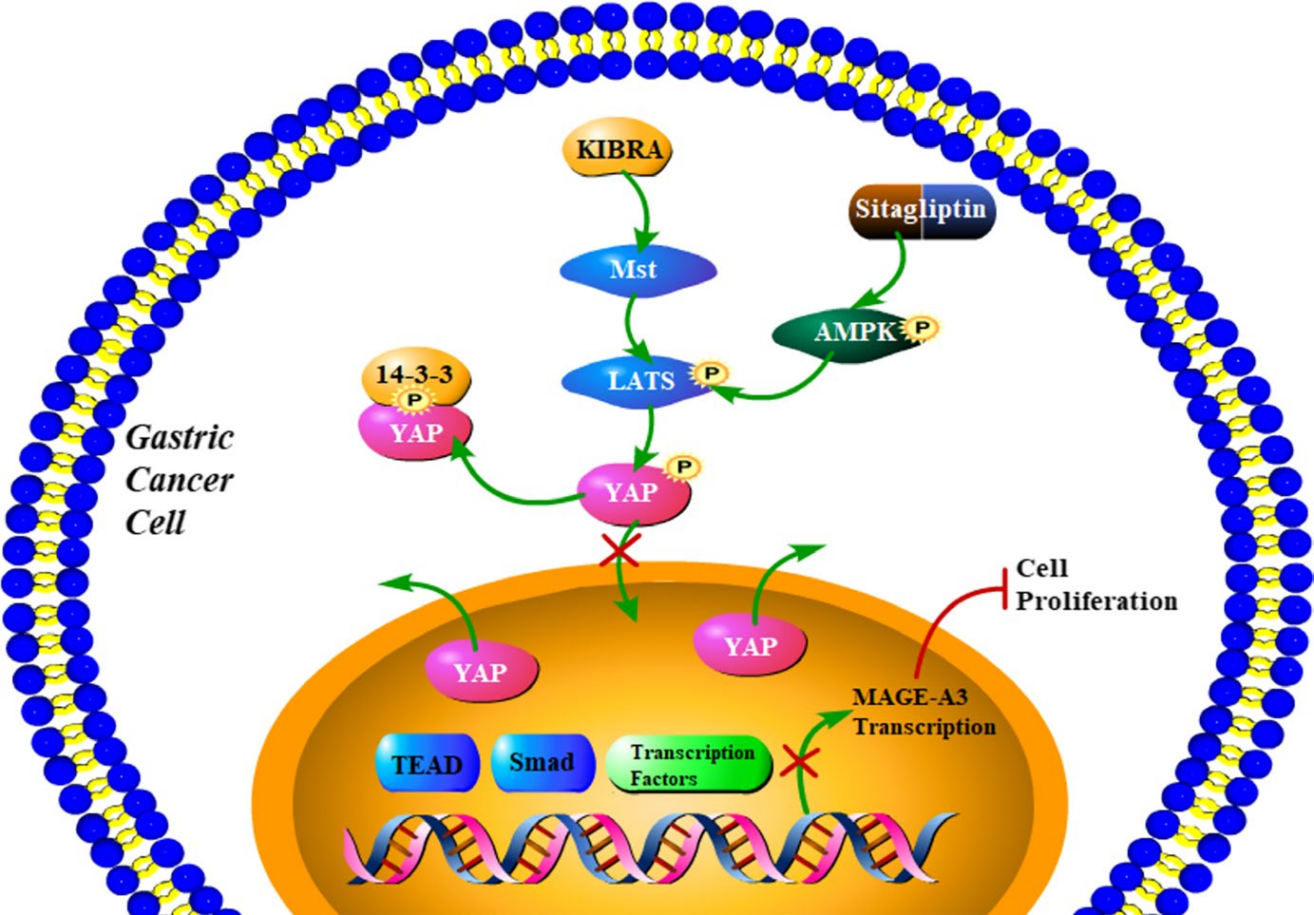
Working model. Sitagliptin affects gastric cancer cells proliferation by suppressing melanoma‐associated antigen‐A3 (MAGE‐A3) expression through Yes‐associated protein (YAP) inactivation

In this study, we confirmed that sitagliptin inhibited the proliferation and clonality of GC cells, and this process was accompanied by an increase in AMPK and YAP phosphorylation levels and a decrease in YAP nuclear localization. In addition, sitagliptin reduced MAGE‐A3 expression via the YAP pathway, which improved the prognosis of GC (Figure 6). Our results indicated that sitagliptin inhibited YAP and MAGE‐A3 by activating AMPK to create a novel therapeutic target for the GC treatment.

## 5 CONCLUSION

In conclusion, our research reveals that AMPK/YAP/MAGE‐A3 pathway plays a key role in regulating sitagliptin's inhibition of GC cell proliferation. These findings will not only deepen our understanding of each molecule, but more importantly, will provide new strategies for the treatment of GC.

## CONFLICT OF INTEREST

The authors have no conflicts of interest to declare.

## AUTHOR CONTRIBUTIONS

All authors provided substantial contributions to this article. Qi Wang, Pan Lu, and Tao Wang participated in experimental design, data collection, and data analysis. Qi Wang wrote the original draft. Qianqian Zheng, and Yan Li participated in the data collation. Sean X. Leng was responsible for reviewing and providing suggestions. Xin Meng, and Biao Wang participated in the conception of the experiment and revised the first draft. Jisheng Xie, and Haiyan Zhang participated in the design of the experiment and made hypothetical comments. All authors read and approved the final version.

## Supporting information

Figure S1

Supplementary Material

## Data Availability

The data in this study are available from the corresponding author upon reasonable request.
