# Supplementary figures and images for: Sitagliptin affects gastric cancer cells proliferation by suppressing Melanoma‐associated antigen‐A3 expression through Yes‐associated protein inactivation
Source: Cancer Med. 2020 Mar 30;9(11):3816–28. doi: 10.1002/cam4.3024 (PMC7286447; doi:10.1002/cam4.3024)

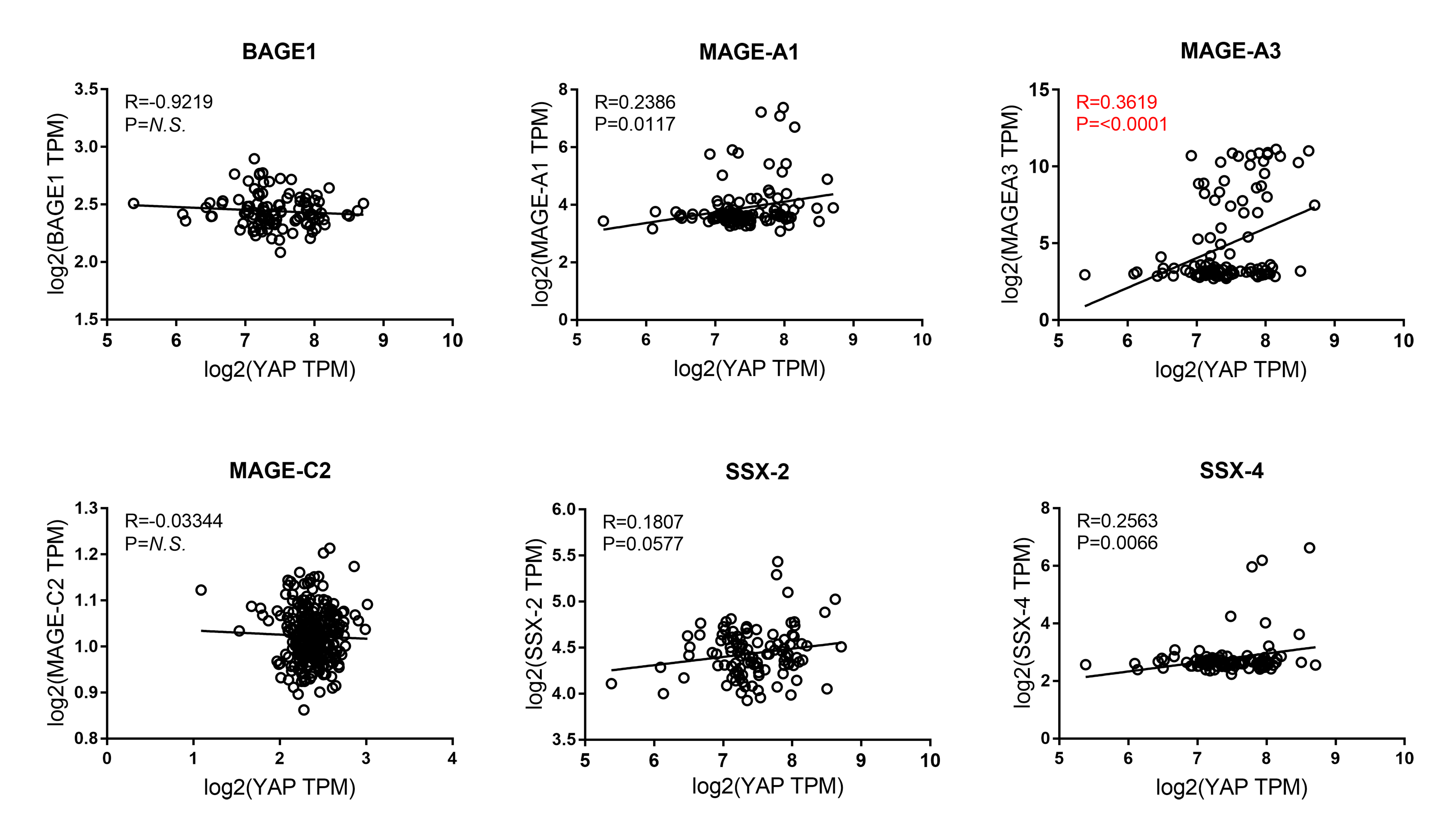

Supplement: Supplementary file 1 — Figure S1 [file CAM4-9-3816-s001.tif]
