# Supplementary material for: Sitagliptin affects gastric cancer cells proliferation by suppressing Melanoma‐associated antigen‐A3 expression through Yes‐associated protein inactivation
Source: Cancer Med. 2020 Mar 30;9(11):3816–28. doi: 10.1002/cam4.3024 (PMC7286447; doi:10.1002/cam4.3024)
Supplement: Supplementary file 2 — Supplementary Material [file CAM4-9-3816-s002.doc]

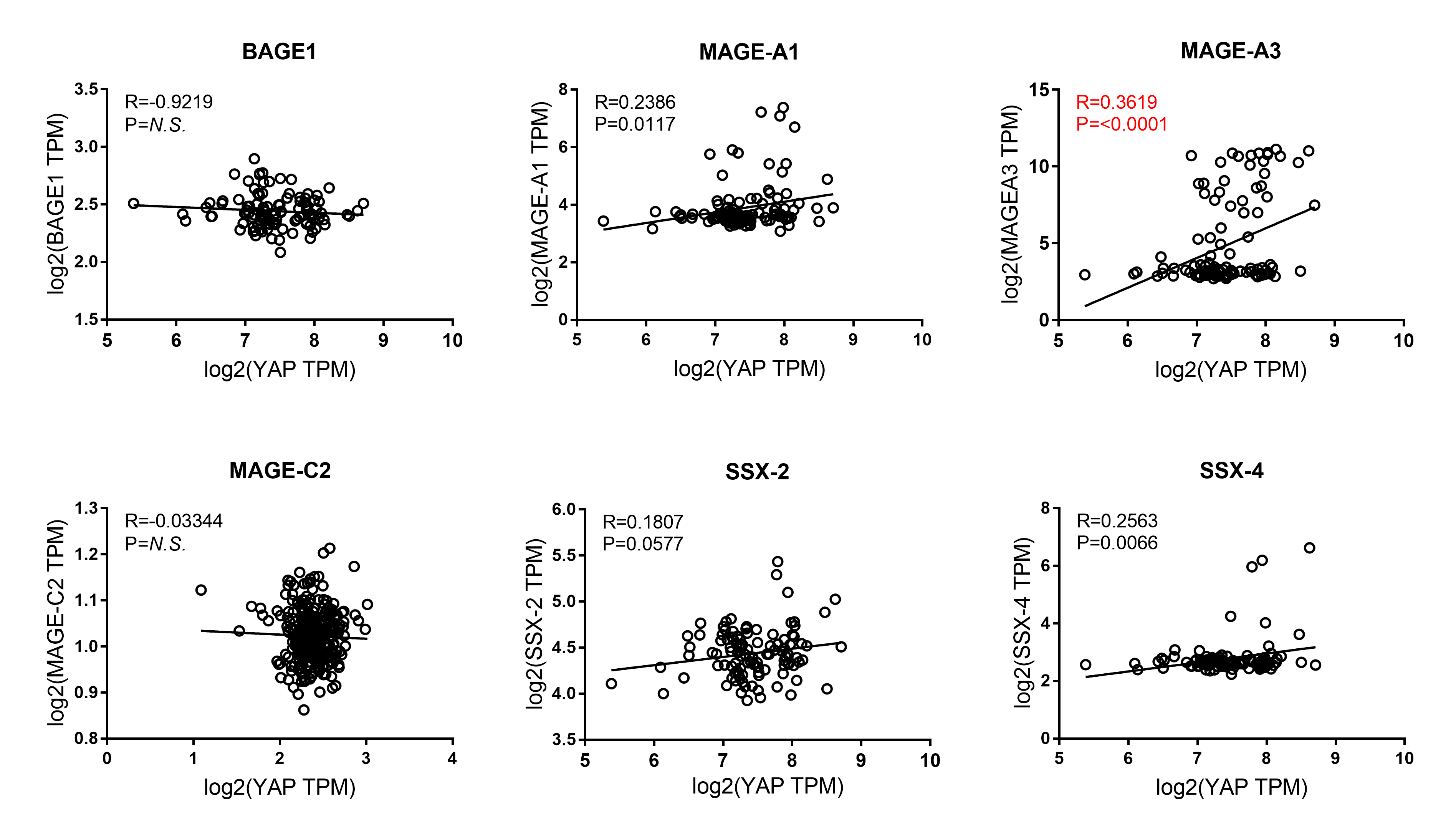
**Supplemental Figure 1** The correlation analysis between BAG1, MAGE-A1, MAGE-A3, MAGE-C2, SSX-2, and SXX-4 genes and YAP.
